# Diurnal variation in cholesterol 7α-hydroxylase activity is determined by the -203A>C polymorphism of the *CYP7A1* gene

**DOI:** 10.3325/cmj.2016.57.111

**Published:** 2016-04

**Authors:** Miluše Vlachová, Tereza Blahová, Věra Lánská, Martin Leníček, Jan Piťha, Libor Vítek, Jan Kovář

**Affiliations:** 1 Institute for Clinical and Experimental Medicine, Prague, Czech Republic; 2 Institute of Clinical Biochemistry and Laboratory Diagnostics, 1st Faculty of Medicine, Charles University in Prague, Prague, Czech Republic; 3 4th Department of Internal Medicine, 1st Faculty of Medicine, Charles University in Prague, Prague, Czech Republic

## Abstract

**Aim:**

To determine whether the promoter polymorphism -203A>C of cholesterol-7α-hydroxylase encoding gene (*CYP7A1*) affects diurnal variation in CYP7A1 enzyme activity.

**Methods:**

The study included 16 healthy male volunteers – 8 homozygous for -203A and 8 homozygous for the -203C allele of *CYP7A1*. Three 15-hour examinations (from 7am to 10pm) were carried out for each of the participants: after one-day treatment with cholestyramine; after one-day treatment with chenodeoxycholic acid (CDCA); and a control examination without any treatment. The plasma concentration of 7α-hydroxy-4-cholesten-3-one (C4), a marker of CYP7A1 activity, was determined in all the experiments at 90-min intervals.

**Results:**

CYP7A1 activity was up-regulated after treatment with cholestyramine and suppressed after treatment with CDCA. There were no differences between -203A and -203C allele carriers in the response of enzyme activity to both drugs. In the control experiment, -203A allele carriers displayed diurnal variation in enzyme activity, whereas CYP7A1 activity did not change in -203C allele carriers. These results were confirmed by modeling the dynamics of C4 using polynomial regression.

**Conclusion:**

The promoter polymorphism of the *CYP7A1* gene has a pronounced impact on diurnal variation in CYP7A1 activity.

Cholesterol 7α- hydroxylase (CYP7A1) is the key regulatory enzyme of the classic bile acid biosynthetic pathway. Its activity is subjected to complex feedback regulation in order to maintain the bile acid pool (1,2). Enzyme activity has been shown to exhibit a distinct diurnal variation in rodents (3) and humans (4). The study in humans took advantage of the fact that CYP7A1 activity in the liver correlated with concentration of 7α-hydroxy-4-cholesten-3-one (C4) in plasma, which can be thus used as a reliable marker of CYP7A1 activity (5,6). The same authors demonstrated that there were two peaks in enzyme activity, the first at midday and the second around 10pm (4). In our previous study in healthy volunteers carried out between 7am and 10pm, we reproduced their findings for the midday peak (7). However, we noted that enzyme activity displayed very high inter-individual variability.

Several polymorphisms have been identified in the regulatory regions of the *CYP7A1* gene. One of these polymorphisms, -203A>C (rs3808607), has been shown to affect cholesterolemia and responsiveness of cholesterolemia to diet as well as drug treatment (8-13). The mechanisms involved have not yet been explained and it is still not clear whether this promoter polymorphism can also affect diurnal variation in enzyme activity. Therefore, in this case-control study, we investigated whether the course of CYP7A1 diurnal activity differed between homozygous young healthy male carriers of -203A and -203C alleles of the *CYP7A1* gene. We also analyzed the effect of short-term chenodeoxycholic acid (CDCA) and cholestyramine (a bile acid sequestrant) treatments on CYP7A1 activity throughout the day. These drugs down-regulate and up-regulate CYP7A1 activity, respectively.

## Participants and methods

### Participants and study design

Sixteen healthy male volunteers – 8 homozygous for the -203A *CYP7A1* allele and 8 homozygous for the -203C allele – were recruited from among the personnel of both participating institutions. Both groups were matched with respect to age (mean±SD, 25.7 ± 3.4 vs 25.3 ± 3.8 years, respectively). The study design was exactly the same as in our previous study (7), consisting of three daylong examinations. One of these examinations served as a control examination (without any drug intervention), while the other two were used to investigate the effect of short-term administration of cholestyramine (Questran®, Bristol-Myers Squibb, Prague, Czech Republic, 16 g/d) and chenodeoxycholic acid (CDCA; Chenofalk®, Dr Falk Pharma GmbH, Freiburg, Germany, 1-1.5 g/d depending on the weight of the participant). One day before each of these examinations (Day 0), the first blood sample was drawn at 7am, after which participants received food for the whole day to standardize their intake before the study. On the day of examination (Day 1), the first blood sample was drawn again at 7am and the blood samples were then collected at 90-min intervals for 15 h until 10pm. Again, participants received food for the whole day and were required to eat at exactly defined intervals (breakfast at 7.15am, snack at 9.45am, lunch at 12.30pm, snack at 3.30pm, and dinner at 5.30pm). The amount of food was calculated to cover their energy requirements – ~ 160 kJ/kg/d – and the diet was relatively low in fat (25% of energy intake). When the examination included drug administration, the drugs were given on Day 0 and Day 1. Cholestyramine was given in two doses on both days – one with breakfast, the other with dinner. Due to differences in pharmacokinetics, CDCA treatment was started with dinner on Day 0, and on Day 1 was given in two doses at the same time as cholestyramine. The order of the examinations was randomized and carried out at three-week intervals at a minimum. The study protocol adhered to the principles of the Declaration of Helsinki, was approved by the Ethics Committee of the Institute for Clinical and Experimental Medicine, and all of the participants gave their informed consent.

### Genotyping and biochemical analyses

Determination of the -203A>C genotype of CYP7A1 was carried out as described earlier (14). Plasma cholesterol and triglyceride (TG) concentrations were determined using enzymatic kits from Roche Diagnostics GmbH, Mannheim, Germany. Concentration of C4 was determined by HPLC as described earlier (15).

### Statistics

ANOVA for repeated measures was used to determine whether the concentrations of cholesterol, TG, and C4 varied throughout the experiments. ANOVA for repeated measures with one grouping factor (genotype) was used to determine whether there were any differences in the course of the concentration changes between carriers of -203A and -203C alleles. The data for analysis were logarithmically transformed if they did not pass the Shapiro-Wilk normality test. However, ANOVA results for logarithmically transformed and nontransformed data were comparable. Presented results of ANOVA are from nontransformed data. The dynamics of C4 concentration changes in the control experiment was modeled using polynomial regression of order 5. The JMP 10.0.0 statistical software (SAS Institute, Inc., Cary, NC, USA) was used for analyses. *P*-value lower than 0.05 was considered statistically significant.

## Results

Homozygous carriers of A and C allele did not differ in plasma cholesterol (4.70 ± 0.91 vs 4.73 ± 0.65 mmol/L, respectively, *P* = 0.941, *t* test) and plasma TG levels (1.78 ± 1.03 vs 1.28 ± 0.39 mmol/L, respectively, *P* = 0.231, *t* test). The -203C allele carriers had significantly increased BMI (27.2 ± 3.3 vs 23.4 ± 3.5 kg/m^2^, *P* = 0.048, *t* test).

As expected, CYP7A1 activity – estimated on the basis of C4 concentration measurement at 7am – rose several-fold after one-day treatment with cholestyramine, dropped by a half after one-day treatment with CDCA, and did not change in the control experiment in all participants (Table 1).

**Table 1:** Fasting concentrations and areas under curve (AUC) of cholesterol, triglyceride (TG), and 7α-hydroxy-4-cholesten-3-one (C4) in homozygous -203A and -203C *CYP7A1* allele carriers (mean ± standard deviation). 0 – control experiment, CDCA – experiment with short-term treatment using chenodeoxycholic acid, Q – experiment with short-term treatment using cholestyramine

		-203AA carriers			-203CC carriers		
		0	CDCA	Q	0	CDCA	Q
**Cholesterol**	**Day 0** **[mmol/L]**	4.69 ±1.08	4.88 ±0.95	4.83 ±0.96	4.70 ±0.65	4.44 ±0.75	4.81 ±0.44
	**Day 1** **[mmol/L]**	4.56 ±1.10	4.98 ±1.09	4.89 ±1.10	4.71 ±0.62	4.63 ±0.61	4.44*** ±0.42
	**AUC** [mmol*h/L]	65.7 ±15.6	70.2 ±14.6	67.9 ±15.0	68.0 ±9.2	67.7 ±9.4	63.2 ±7.0
**TG**	**Day 0** **[mmol/L]**	1.48 ±0.84	1.58 ±0.82	1.71 ±1.26	1.54 ±0.65	1.40 ±0.73	1.33 ±0.41
	**Day 1** **[mmol/L]**	1.39 ±0.82	1.66 ±0.96	1.65 ±0.85	1.70 ±1.03	1.70 ±0.72	1.85* ±0.65
	**AUC** [mmol*h/L]	24.9 ±13.3	28.9 ±15.0	24.0 ±12.9	29.0 ±16.9	27.4 ±9.2	28.1 ±14.3
**C4**	**Day 0** **[μg/L]**	16.0 ±9.1	19.9 ±13.2	13.9 ±8.7	22.9 ±16.6	36.1 ±32,8	28.2 ±21.1
	**Day 1** **[μg/L]**	20.3 ^a,b^ ±15.9	9.6 ^b,**^ ±9.6	70.2 ^a,***^ ±27.3	24.7 ^d,e^ ±23.1	15.6 ^e,**^ ±18.8	111.3 ^d,**^ ±70.1
	**AUC** [μg*h/L]	326 ^b^ ±94	131 ^c^ ±65	1317 ^a^ ±312	270 ^e^ ±168	112 ^e^ ±71	1830 ^d^ ±808

*,**,***P < 0.05, *P* < 0.01, *P* < 0.001 for Day 1 vs Day 0; ^a^,^b^,^c^ the same letters are assigned to the experiments that do not differ in -203A allele carriers where differences between experiments are detected by ANOVA; ^d^,^e^,^f^ the same letters are assigned to the experiments that do not differ in -203C allele carriers, where differences between experiments are detected by ANOVA.

**Figure 1:**
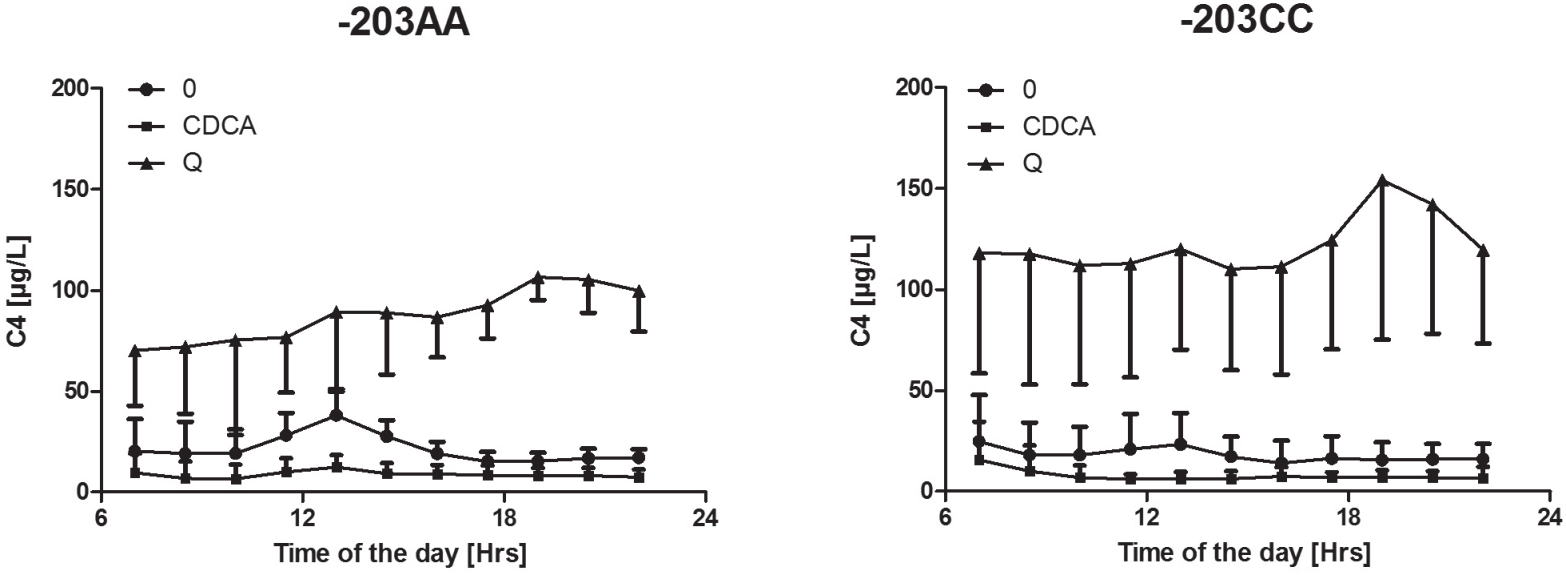
The course of 7α-hydroxy-4-cholesten-3-one (C4) concentrations throughout Day 1 after short-term administration of cholestyramine and CDCA and in the control experiment. 0 – control experiment, CDCA – experiment with short-term treatment using chenodeoxycholic acid, Q – experiment with short-term treatment using cholestyramine; -203AA, -203CC – homozygous carriers of the -203A and -203C allele of the *CYP7A1* gene, respectively.

During the CDCA treatment, there were no changes in plasma C4 concentrations on the day of the study in both -203A and -203C allele carriers (Figure 1) (*P* = 0.373 and 0.208, respectively). Importantly, no effect of the genotype on the course of C4 concentrations was detected (*P* = 0.221).

During the cholestyramine treatment, C4 concentrations significantly increased during the day in -203A allele carriers (*P* = 0.004). A similar but not significant trend was observed in -203C allele carriers (*P* = 0.074). However, the genotype had no effect on the course of C4 concentrations throughout the day (*P* = 0.659).

**Figure 2:**
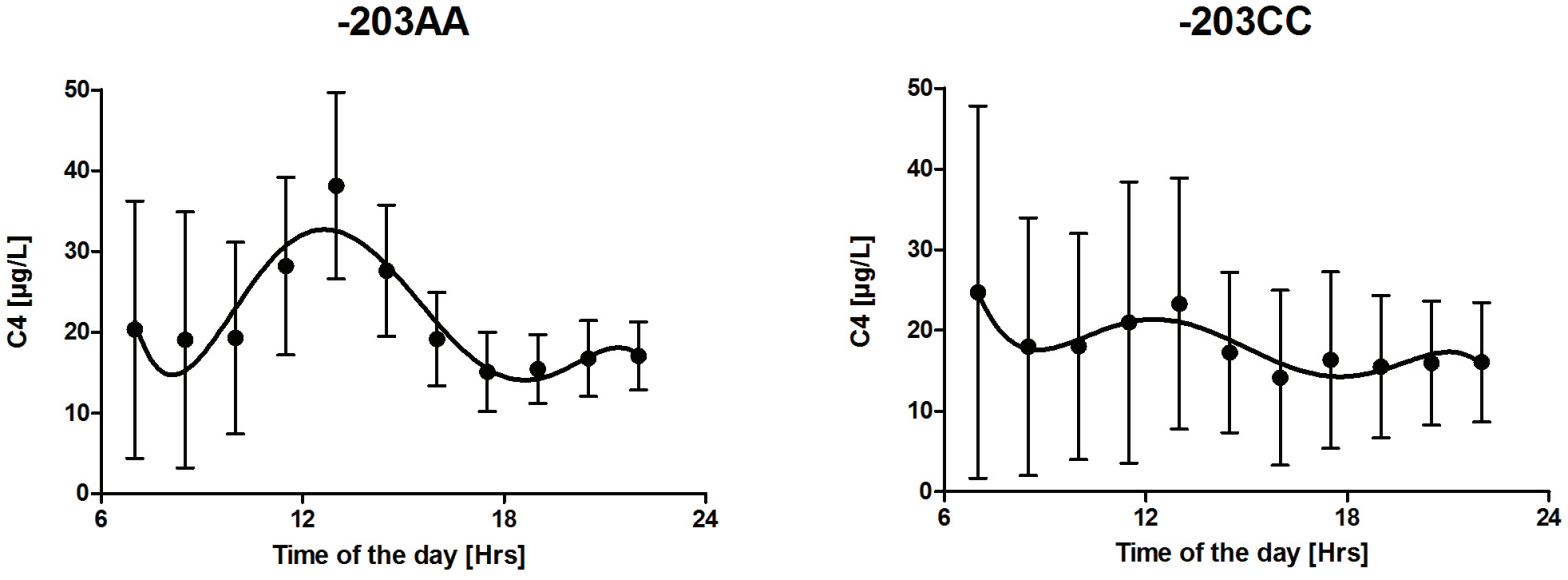
The course of 7α-hydroxy-4-cholesten-3-one (C4) concentrations throughout Day 1 as modeled using polynomial regression of order 5. -203AA, -203CC – homozygous carriers of the -203A and -203C allele of the *CYP7A1* gene, respectively.

In the control experiment, plasma C4 concentrations significantly varied during the day in -203A allele carriers (*P* = 0.001) with a peak around 1pm. On the other hand, C4 concentrations did not change during the day in -203C allele carriers. Moreover, there was a trend for the effect of the genotype on the course of C4 concentrations (*P* = 0.092). These results suggest that there may be differences in the diurnal variation in CYP7A1 activity between homozygous -203A and -203C allele carriers. To test such a hypothesis, we modeled the dynamics of C4 concentration changes using fifth order polynomial regression (Figure 2, Table 2). The regression equation coefficient of order 2 significantly differed from zero only in -203A allele carriers, but not in those carrying the -203C allele. Importantly, the coefficients of the second and fourth order of corresponding regression equations were different between carriers of -203A and -203C alleles.

**Table 2:** Coefficients of the polynomial regression equation (a_0_, a_1_, a_2_, a_3_, a_4_, a_5_) of order 5 for the relationship between 7α-hydroxy-4-cholesten-3-one (C4, µg/L) concentration and time of day (t, hours) in -203A and -203C allele carriers. C4 = a_0_ + a_1_*(t-7) + a_2_*(t-14.5)^2^ + a_3_*(t-14.5)^3^ + a_4_*(t-14.5)^4^ + a_5_*(t-14.5)^5^. Estimates are mean (SE).

	-203AA carriers	-203CC carriers
Coefficient	Coefficient estimate	Coefficient estimate
a_0_	60.20 (8.96)**	33.19 (4.36)***
a_1_	-4.258 (1.158)*	-1.918 (0.563)*
a_2_	-0.6564 (0.2174)*	-0.1563 (0.1058)†
a_3_	0.2135 (0.0733)*	0.1021 (0.0357)*
a_4_	0.0088 (0.0037)	0.0032 (0.0018)†
a_5_	-0.0025 (0.0010)	-0.0014 (0.0005)*

*,**,***P < 0.05, *P* < 0.01, *P* < 0.001 for the coefficient estimate being equal to zero.

†*P* < 0.05 for coefficient estimates being equal for homozygous carriers of the -203A and -203C alleles. The equation is valid for the interval between 7am until 10pm.

CDCA treatment had no effect on cholesterol and TG concentrations for all of the participants. Administration of cholestyramine resulted in an 8% decrease in cholesterol concentration and a 39% increase in triglyceridemia in homozygous -203C allele carriers. No such effect was observed in -203A allele carriers. However, the AUCs of both cholesterol and TG on Day 1 did not differ between -203A and -203C allele carriers (Table 1).

## Discussion

This small study of healthy volunteers showed that homozygous participants carrying the -203A allele of the *CYP7A1* displayed diurnal variation in CYP7A1 activity, peaking at midday. No diurnal variation in CYP7A1 activity was observed in homozygous carriers of the -203C allele.

Such findings were further corroborated by modeling the dynamics of C4 concentration changes using polynomial regression of order 5. Importantly, we found that the coefficients of the second and fourth order (a_2_ and a_4_) of the corresponding regression equations were different in the carriers of -203A and -203C alleles. It should be pointed out that only functions with nonzero coefficients of odd order (2 and 4 in our case) display the presence of a peak, while coefficients of even order do not. This strongly supports the idea that the course of C4 concentrations differs between homozygous carriers of the two alleles – the peak of CYP7A1 activity can be observed only in A allele carriers.

It has been shown that CYP7A1 activity in humans has two peaks (4) – one in the early afternoon and the second before midnight – but due to the design of our study, we can make conclusions only about the first peak. Interestingly, there is no clear mechanistic explanation for the midday peak. It has been demonstrated that such a peak appears both in participants who eat normally and those who are fasting before midday (4). Such an increase is thus unlikely to be associated with food intake. Interestingly, CYP7A1 activity then decreases rapidly only in subjects who eat normally and not in those who are fasting. This may suggest that increased intrahepatic flux of bile acids, and especially intestinal fibroblast growth factor-19 (FGF-19) secretion, after a meal are involved in dampening the peak of CYP7A1 activity (16).

Using a dual luciferase reporter assay with promoter fragments (-716 to +14) of both variants of the *CYP7A1*, we confirmed the previous findings that in HepG2 cells the -203C allele was expressed five times more than the -203A allele (17). However, we did not see any differences in the effects of bile acids, cortisol and dexamethasone, insulin, and peroxisome proliferator-activated receptor α (PPARα) agonists (fenofibrate and WY-14643) on the expression of both promoter variants (Vlachová M, Blahová T, unpublished data). This may suggest that factors other than those associated with food intake may play a role in the regulation of CYP7A1 activity throughout the day. Furthermore, we tried to find whether any transcription factor binds differently to both variants of the promoter surrounding -203 position using *in silico* analysis (Transcription Element Search System, Vlachová M, unpublished data). Except of putative new binding site for a glucocorticoid receptor [AGAA_-203_CT] in -203A allele (which does not seem to be functional based on dual luciferase data), no other differences between the promoter variants were found. No differences in the presence of putative binding sites were found also in five other polymorphisms (rs1023652, rs1023649, rs3903445, and rs3824260) that are in a tight linkage disequilibrium with -203A>C variant (rs3808607).

The diurnal variation in CYP7A1 activity and *Cyp7a1* gene expression has been extensively studied in mice and rats and it was demonstrated that *Cyp7a1* expression in mice was controlled by several clock genes (18-22). It remains to be determined whether clock genes also play a role in the regulation of bile acid synthesis in humans.

Given that some participants do not display diurnal variation, it is surprising that this has remained unnoticed so far. This is likely due to the small sample size of studies investigating circadian variation in CYP7A1 activity (4,16), and it cannot be excluded that -203C allele carriers were not studied. This can even suggest that the effect of more prevalent -203A allele is dominant and heterozygous carriers display diurnal variation in CYP7A1 activity.

It must be also stressed that the -203A>C polymorphism may not be the one responsible for observed differences in circadian changes in CYP7A1 activity. It has been demonstrated that this polymorphism is in close linkage disequilibrium with several other polymorphisms in the *CYP7A1* (23). In the Caucasian population (the only population in which studies of diurnal variation in CYP7A1 have been carried out), the -203A and -203C alleles are considered to be markers of haplotype blocks spanning 14 kb from the proximal promoter to the 3′-downstream of the *CYP7A1*. Therefore, either of the polymorphisms included in these haplotype blocks may be responsible for the observed differences in diurnal variation in the enzyme activity.

The fact that C allele carriers cannot increase the CYP7A1 diurnal activity might explain their hyperresponsiveness to dietary cholesterol and saturated fat as observed in previous studies (10-12). They might be unable to eliminate the excess of dietary cholesterol through its transformation into bile acids as efficiently as A allele carriers, and their cholesterolemia might be augmented.

The major limitation of this study, apart from the small sample size, is the lack of night blood sampling. In future studies, 24-hour monitoring and inclusion of heterozygous individuals should be undertaken. It should be kept in mind that C4 is only a surrogate marker of CYP7A1 activity – however, it is hard to overcome such a limitation in human studies. Additionally, -203C carriers in our cohort had higher BMI.

In conclusion, we demonstrated that the -203A allele of the *CYP7A1* was associated with pronounced diurnal variation in CYP7A1 activity, whereas the -203C allele was not. It remains to be determined whether differences in diurnal variation in enzyme activity between carriers of the -203A and -203C alleles can explain the differential effects of these variants on cholesterolemia and its responsiveness to diet.
